# Molecular sampling limits of ctDNA detection in clinical plasma samples

**DOI:** 10.1016/j.jlb.2026.100480

**Published:** 2026-07-04

**Authors:** Alexander Gamisch

**Affiliations:** Independent Researcher, Salzburg, 5020, Austria

**Keywords:** Circulating tumor DNA, Cell-free DNA, Liquid biopsy, Genome equivalents, Limit of detection, Variant allele fraction

## Abstract

Circulating tumor DNA (ctDNA) assays are commonly described by fixed variant allele fraction (VAF)–based limits of detection. However, such metrics overlook a fundamental constraint: the finite number of analyzable DNA molecules in an individual plasma sample. As a result, nominal assay sensitivity may overestimate what is physically achievable in routine clinical specimens. Genome-equivalent (GE) distributions from three independent liquid-biopsy cohorts comprising 5238 plasma samples were integrated with a Poisson-based sampling model to estimate input-limited detectability for single-locus variants. For each sample, the lower limit of detection was defined as the minimum VAF associated with a 95% probability of observing at least 1, 3, 5, or 10 mutant molecules. GE input varied widely (median 5531; interquartile range 2784–13,060). Detection at 1% VAF was theoretically achievable for nearly all samples, but performance declined sharply at lower VAFs and with increasing evidentiary thresholds. At 0.1% VAF, 72% of samples supported detection of at least one mutant molecule, compared with 46%, 35%, and 21% for thresholds of at least 3, 5, and 10 molecules. At 0.01% VAF, fewer than 10% of samples met any detection criterion. These findings indicate that many clinical plasma samples are unlikely to support reliable single-locus detection at very low VAFs, independent of assay design. Sample-aware interpretation of ctDNA results that accounts for molecular input is therefore warranted.

## Graphical abstract

**Figure:**
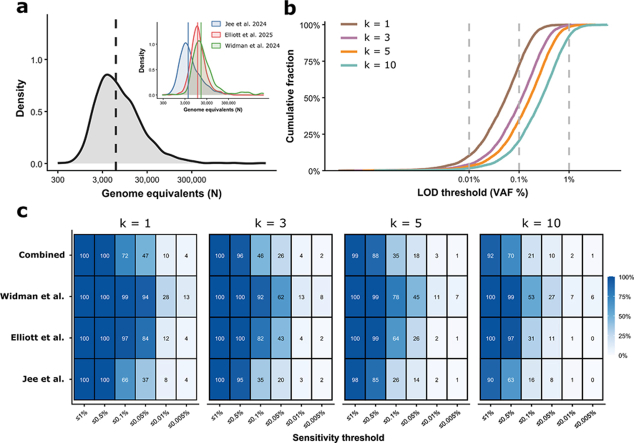

## 1 Introduction

Circulating tumor DNA (ctDNA) analysis has become an important component of precision oncology by enabling minimally invasive detection of tumor-derived genomic alterations for molecular profiling, therapy selection, and disease monitoring. In clinical practice, plasma genotyping can provide actionable results within a short turnaround time and is particularly valuable when tissue is unavailable, insufficient, risky to obtain, or delayed [1,2]. At the same time, tissue-based analysis remains the reference standard for comprehensive molecular characterization in most settings. Accordingly, negative plasma findings must be interpreted in the context of the biological and technical limits inherent to ctDNA analysis [1,2].

One major and often underappreciated limitation is the finite and highly variable amount of cell-free DNA (cfDNA) recovered from plasma [[1], [2], [3], [4]]. Even under favorable conditions, the number of genome equivalents available for analysis is modest in relation to the very low variant allele fractions (VAFs) that are often clinically relevant. As a result, the sensitivity of a ctDNA assay is constrained not only by sequencing depth, error suppression, and bioinformatic performance, but also by the absolute number of input molecules physically present in the specimen [1,[5], [6], [7], [8]]. This sampling constraint is particularly important for single-locus detection, where the presence or absence of only a few mutant molecules may determine whether a variant is observed at all.

A related challenge is that the fraction of tumor-derived DNA within total cfDNA is frequently very low, especially in early-stage disease, minimal residual disease, emerging relapse, and treatment monitoring. In such settings, clinically relevant variants may occur at VAFs far below 1%, often approaching or falling below the molecular detection range that can realistically be supported by clinical plasma input [[9], [10], [11]]. Consequently, even analytically highly sensitive assays may fail to detect variants simply because too few mutant molecules are present in the analyzed aliquot.

Prior work has established Poisson-based and related probabilistic frameworks for modeling the relationship between molecular input, variant allele fraction, and the probability of detecting low-frequency variants in liquid biopsy specimens [1,5,6,8]. These contributions have been instrumental in articulating the theoretical basis of input-limited detectability and in deriving minimum genome-equivalent requirements for single-locus ctDNA detection. However, these frameworks have rarely been applied to large real-world cfDNA input distributions from routine clinical samples. In particular, it remains unclear how often clinical plasma samples contain enough genome equivalents to support detection at commonly cited low-VAF thresholds, and how strongly this depends on the number of mutant molecules required for variant calling.

This gap has direct clinical consequences. Plasma assay performance is routinely communicated as a fixed, nominal VAF-based limit of detection, a metric derived under optimized analytical conditions that does not account for the molecular information content of individual specimens. When applied to clinical plasma samples, which vary widely in cfDNA yield and are frequently processed under constraints that reduce effective assay input, such fixed sensitivity claims may substantially overestimate what is physically achievable in a given patient specimen. Greater transparency regarding the distinction between analytical assay sensitivity and sample-specific detectability may improve interpretation of negative liquid-biopsy findings, reduce overinterpretation of false-negative results, and support more realistic expectations of plasma genotyping across clinical settings.

Here, genome-equivalent distributions from three liquid-biopsy cohorts comprising 5238 plasma samples were integrated with a Poisson-based framework to quantify how clinical plasma input constrains single-locus ctDNA detectability. The analysis explicitly models the dependence of input-limited detectability on the minimum number of mutant molecules required for variant calling, thereby translating an assay-design parameter that is often only implicit into a population-level estimate of detection probability across real-world specimens. In addition, an interactive web application is provided to enable sample-specific estimation of theoretical detectability under user-defined input and evidentiary assumptions.

## 2 Methods

Genome-equivalent (GE) distributions from three published liquid-biopsy datasets [[12], [13], [14]] were harmonized by converting cfDNA quantities to haploid genome equivalents. Where directly comparable plasma-volume information was unavailable, values were standardized to a 4-mL plasma-equivalent as a pragmatic reference input consistent with commonly recommended minimum plasma volumes for clinical cfDNA analysis [15] and correspond to approximately 8–10 mL of whole blood, consistent with a single standard EDTA or cell-stabilizing collection tube [7,15]. Sensitivity analyses spanning 1 to 12 mL plasma equivalents are provided to contextualize results across a range of collection volumes encountered in clinical practice. Expected mutant-molecule counts were modeled as a function of GE input and variant allele fraction (VAF), and the probability of observing at least *k* mutant molecules was estimated using a Poisson framework. For each sample, the theoretical lower limit of detection was defined as the minimum VAF associated with a 95% probability of observing at least *k* mutant molecules (*k* = 1, 3, 5, and 10). The criterion *k* = 1 reflects the absolute molecular minimum, whereas *k* = 3 approximates scenarios in which at least three independent positive observations, such as three positive droplets, three independent UMI families, or three non-duplicate mutant-supporting reads, are required for a confident variant call [5,16]. The stricter thresholds *k* = 5 and *k* = 10 model increasingly conservative evidence requirements for robust detection (cf. Heitzer et al. [6]). In addition, the proportion of samples achieving LODs at or below predefined thresholds as inspired by commonly reported detection limits of clinical diagnostic ctDNA profiling assays (i.e. 1%, 0.5%, 0.1%, 0.05%, 0.01%, and 0.005% VAF; see Refs. [2,17]) was calculated for each study and for the pooled dataset. Additional conversion details, sensitivity analyses, and implementation parameters are provided in the Supplementary Methods. All analyses and visualizations were performed in R v. 4.5.3 [18] using the functionalities of the packages *ggplot2* [19], *dplyr* [20], *tidyr* [21], *readr* [22], *patchwork* [23], and *scales* [24].

## 3 Results

### 3.1 Genome equivalent distributions

**Fig. 1:**
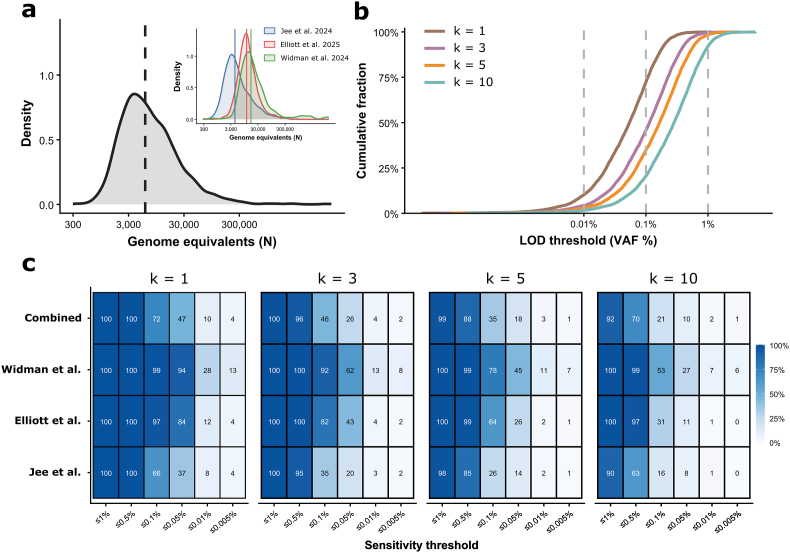
**Molecular constraints on ctDNA detection in clinical plasma samples under a Poisson sampling model**. Genome equivalent (GE) distributions from three independent cfDNA datasets were used to model theoretical single-locus detection limits under Poisson sampling. The expected number of mutant molecules was defined as λ = N_eff_ × VAF, where N_eff_ denotes the effective number of genome equivalents and VAF the variant allele frequency. For this analysis, the effective input fraction was set to *f* = 1, such that *N*_*eff*_*= N*. Limits of detection (LOD) were defined as the VAF at which the probability of observing at least *k* mutant molecules reached 95%, i.e. *P*(*X* ≥ *k*) = 0.95. **(a)** Density distributions of available genome equivalents in the combined dataset and in the three individual studies (inset); vertical lines indicate the median GE value for each distribution. **(b)** Empirical cumulative distribution functions of Poisson-based LODs across all samples for detection thresholds requiring at least 1, 3, 5, or 10 mutant molecules (k = 1, 3, 5, 10). Dashed vertical lines indicate selected reference thresholds (0.01%, 0.1%, and 1% VAF). **(c)** Heatmaps summarizing the percentage of samples in each study, as well as in the combined dataset, predicted to achieve predefined sensitivity thresholds (≤1%, ≤0.5%, ≤0.1%, ≤0.05%, ≤0.01%, and ≤0.005%) under each Poisson-based detection criterion.

Across the pooled dataset (n = 5238), median input was 5531 GE (IQR, 2784–13,060), with marked variability across the three source datasets (Fig. 1a; Supplementary Fig. S1). Cohort-specific GE distributions and conversion details are provided in the Supplementary Methods and Supplementary Table S1.

### 3.2 Real-world plasma samples frequently lack sufficient mutant molecules at low VAFs

Across datasets, Poisson-based detectability was strongly constrained by input DNA quantity. Most samples supported detection at 1% VAF, whereas detectability declined markedly at lower VAFs and with increasing mutant-molecule thresholds (Fig. 1b and c). In the combined dataset, 72% of samples achieved a Poisson-based LOD of ≤0.1% for *k* = 1, compared with 46% for *k* = 3, 35% for *k* = 5, and 21% for *k* = 10. At ≤0.05% VAF, the corresponding proportions were 47%, 26%, 18%, and 10%, respectively. At ≤0.01% VAF, only 10%, 4%, 3%, and 2% of samples met the threshold. Although Widman et al. showed the most favorable input profile and Jee et al. the least favorable, the same overall pattern was observed across all datasets (Fig. 1c).

**Fig. 2:**
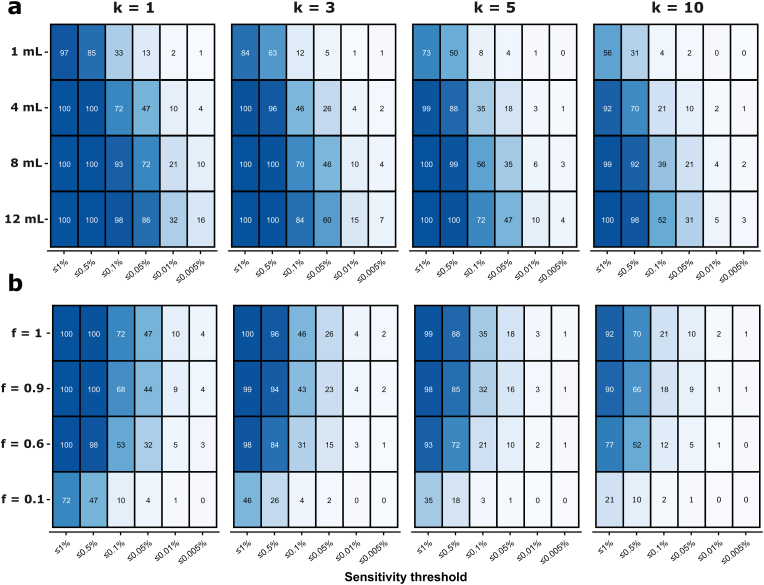
**Effects of plasma input volume and effective assay input on Poisson-based ctDNA detectability.** Heatmaps show the percentage of samples in the pooled dataset predicted to achieve predefined Poisson-based lower limits of detection (LOD) for single-locus ctDNA variants. LOD was defined as the minimum variant allele fraction (VAF) associated with a 95% probability of observing at least *k* mutant molecules (*k* = 1, 3, 5, or 10). Columns indicate target sensitivity thresholds (≤1%, ≤0.5%, ≤0.1%, ≤0.05%, ≤0.01%, and ≤0.005% VAF). **(a)** Modeled plasma inputs of 1, 4, 8, and 12 mL, assuming proportional scaling of genome-equivalent input with plasma volume and full assay input (*f* = 1). **(b)** Modeled effective assay input fractions of *f* = 1, 0.9, 0.6, and 0.1 for the 4 mL reference scenario, where *N*_eff_ = *N* × *f*.

Sensitivity analyses across theoretical plasma inputs of 1, 4, 8, and 12 mL showed the expected improvement in achievable Poisson-based detectability with increasing input volume, but did not alter the overall pattern of sharply reduced support for very low-VAF detection under more stringent mutant-molecule thresholds (Fig. 2a). Likewise, reduced effective input fractions led to the expected deterioration in theoretical detectability across all thresholds. Thus, lower effective assay input amplified, rather than altered, the main finding that molecular input strongly constrains low-frequency single-locus ctDNA detection (Fig. 2b). To facilitate practical application in clinical and research workflows, the model was implemented as an interactive web application that enables sample-specific estimation of theoretical ctDNA detectability based on user-defined assay input parameters. An example interface is shown in Supplementary Fig. S2. The web application source code has been included in the Supplementary Material.

## 4 Discussion

This study examined how genome equivalent (GE) input constrains the practical detectability of ctDNA variants in clinical plasma samples. Integration of real-world GE distributions from three independent datasets with a Poisson-based sampling model showed that sensitivity is determined not only by assay characteristics, but also by the number of mutant template molecules physically present in the analyzed specimen. Accordingly, a nominal variant allele fraction (VAF)-based limit of detection does not by itself indicate what is achievable in an individual plasma sample.

A central finding of the analysis is that the practical lower limit of detection for single-locus ctDNA assays is fundamentally bounded by molecular input. Under idealized sampling assumptions, detection with 95% probability can be approximated as a function of total genome equivalents and the minimum number of mutant molecules required for a positive call. While *k* = 1 represents the absolute molecular minimum, stricter requirements such as *k* = 3, 5, or 10 markedly shift the achievable detection range. This distinction is clinically relevant because clinical diagnostic workflows typically require more than a single molecular event to distinguish true variants from sequencing or PCR artifacts and to support confident interpretation [5,16].

Applied to real-world plasma inputs, the clinical implications are clear. Across the pooled cohort, most samples theoretically supported variant detection at 1% VAF, but performance dropped substantially at lower VAFs, particularly under more conservative evidence thresholds. At ≤0.1% VAF, 72% of samples achieved the threshold for *k* = 1, compared with 46%, 35%, and 21% for *k* = 3, 5, and 10, respectively. At ≤0.01% VAF, only a small minority of samples remained compatible with reliable detection under any criterion. These findings indicate that many clinical plasma specimens are unlikely to contain enough mutant molecules to support robust single-locus detection in the very low-VAF range, even before additional technical losses are considered.

This has important consequences for interpretation of negative ctDNA results. In samples with low tumor burden, such as early-stage disease, minimal residual disease, relapse, or treatment monitoring, failure to detect a variant may reflect stochastic molecular undersampling rather than true biological absence. A negative plasma result at very low expected VAF therefore cannot automatically be interpreted as evidence that the target alteration is absent; it may simply have been absent from the analyzed aliquot. These data reinforce the need to distinguish between analytical assay sensitivity under optimized validation conditions and sample-specific detectability in clinical practice. While this distinction applies across the spectrum of liquid-biopsy indications, the constraint is most consequential in low-burden, low-VAF settings such as early-stage disease, MRD, and molecular relapse; in the advanced and metastatic settings that anchor current guideline-endorsed ctDNA testing, molecular inputs and VAFs are generally higher and the present estimates correspondingly conservative. It should also be noted that the present model operates at the level of total cfDNA input and does not account for the additional layer of variability introduced by tumor-specific shedding dynamics. The fraction of ctDNA within total cfDNA, and thus the effective VAF of any given variant, is itself subject to substantial biological variability driven by tumor stage, histology, tumor location, treatment effects, and intra- and inter-tumoral heterogeneity [1,10,17]. In early-stage or low-burden settings, ctDNA may constitute only a minor fraction of total cfDNA, further reducing the expected number of mutant molecules below what would be predicted from GE input alone. The sampling constraints described here therefore represent a lower bound on the complexity of real-world detectability: even if a sample contains sufficient total GEs, the number of tumor-derived molecules at a given locus may still be too low for reliable detection if ctDNA shedding is sparse or intermittent. Molecular input and tumor shedding thus act as compounding, rather than alternative, constraints on ctDNA sensitivity.

A related and often underappreciated issue is that total extracted cfDNA is not equivalent to the DNA actually interrogated by the assay (e.g. Elliott et al. [13]). In practice, only a fraction of recovered material may enter library preparation or PCR because of elution constraints, assay input limits, repeat testing, or parallel analyses (cf. Song et al. [8]). In the model, this is captured by the effective assay input (*N*_*eff*_ *= N × f*), for which *f* = 1was assumed in the primary analysis. In reality, however, *f* is often below 1, meaning that the number of mutant molecules available for detection is lower than suggested by total cfDNA yield alone. Sensitivity analyses with reduced effective input fractions indicate that the primary *f* = 1 scenario represents an optimistic upper-bound approximation of input-limited detectability. Under more restrictive and likely more realistic assumptions, the fraction of samples supporting low-VAF detection decreases further, while the central conclusion remains unchanged (Fig. 2b).

The present framework applies most directly to single-locus (variant-specific) detection of SNVs and indels, where detectability depends on whether sufficient mutant molecules are present for a specific genomic alteration of interest, such as a clinically relevant *KRAS*, *BRAF*, *PIK3CA*, or *ESR1* variant, even when interrogated within a broader multigene genotyping panel, rather than to multilocus MRD assays that aggregate signal across multiple patient-specific variants and are often interpreted as a binary result (e.g., ctDNA present vs absent). Multilocus approaches can partially mitigate sampling limitations by integrating evidence across several variants and thereby increasing the probability of capturing at least some tumor-derived molecules (cf. Heitzer et al. [4]). Nevertheless, even such approaches remain constrained by the same underlying biology, because the total number of ctDNA molecules available in plasma is finite and very low disease burden will still challenge detectability.

An important practical implication of the study is the potential value of sample-specific reporting of ctDNA detection limits, as previously advocated by others [1,16,25]. Genome equivalents provide a simple and transparent proxy for the molecular information content of a plasma sample and can be derived from routinely measured cfDNA quantities. Reporting detectability either as a simple input-based approximation (e.g. *k/N*) or, more precisely, as a sample-specific Poisson-based LOD for a defined evidence threshold could help contextualize negative findings and communicate the effective sensitivity realistically achievable in a given specimen. To facilitate practical application, the model was also implemented as an interactive web application (https://aga1.shinyapps.io/ctdna_lod_app/) that enables intuitive exploration of how molecular input, effective assay input, and mutant-molecule requirements shape the achievable lower limit of detection.

This study also has limitations. First, the model is intentionally simplified and assumes random sampling of mutant molecules according to a Poisson process**,** and does not account for clonal hematopoiesis (CH): somatic mutations in CH-associated genes (e.g. DNMT3A, TET2, ASXL1) can contribute variant signals to plasma cfDNA that are indistinguishable from ctDNA by VAF alone [1,2,4], a specificity-related limitation orthogonal to the sensitivity framework presented here. Second, the datasets included here differ in clinical context, pre-analytical methodology (extraction platform, collection tube, centrifugation protocol), and reporting format (see Supplementary Material). Inter-laboratory pre-analytical variability may contribute to inter-cohort GE differences (Fig. 1a; Supplementary Fig. S1) independently of biological shedding, though the within-cohort consistency of the central finding (Fig. 1c) and sensitivity analyses spanning 1–12 mL plasma input (Fig. 2a) confirm that this does not alter the overall conclusion. Third, the model implicitly assumes that measured GE reflects genuine nucleosome-fragmented cfDNA; gDNA contamination from leukocyte lysis would inflate the GE denominator and dilute tumour VAF [1,2], biasing detectability estimates upward, an effect functionally equivalent to reduced effective input (*f* < 1; Fig. 2b) and thus reinforcing rather than weakening the central conclusion. cfDNA fragment-size and integrity profiling (e.g., electrophoretic sizing) is therefore recommended as a pre-analytical quality criterion for prospective implementations of sample-aware ctDNA reporting. Finally, an additional limitation is that the present framework was not validated against experimentally measured detection rates from an independent cohort. As the model is mathematically derived with no free parameters estimated from the data, its predictions follow directly from Poisson sampling theory and do not require empirical training. Nevertheless, future studies combining measured cfDNA inputs with orthogonal detection assays would be valuable to empirically assess the predictive performance of the proposed framework.

Despite these limitations, the consistency of the observed pattern across all three datasets supports a robust general conclusion: molecular input alone imposes a major and often underrecognized constraint on ctDNA detection at low VAFs. Accordingly, claims of fixed assay sensitivity, particularly in the range of 0.1% VAF and below, should be interpreted in light of the actual number of genome equivalents available for analysis and the evidentiary threshold required for variant calling. Although genome equivalents do not capture all determinants of assay performance, they provide a practical first-order metric of sample-specific detectability. Incorporating such input-aware interpretation into ctDNA reporting may improve the clinical realism of liquid-biopsy results and reduce overinterpretation of negative findings in clinical practice. Overall, the findings support a shift from fixed nominal sensitivity claims toward a more sample-aware interpretation of ctDNA results that explicitly accounts for molecular input constraints.

## Consent to participate

Not applicable.

## Ethics approval

Ethics approval was not required for this study because it involved secondary analysis of previously published, de-identified data from independent studies and did not involve direct contact with human participants, access to identifiable personal data, or collection/use of new human biological specimens.

## Data availability statement

The author declares that the data supporting the findings of this study are available within the paper and its Supplementary Information files. Should any raw data files be needed in another format they are available from the corresponding author upon reasonable request. Source data are provided with this paper.

## Consent for publication

Not applicable.

## Author contributions

AG conducted all aspects of the work and approved the final manuscript.

## Declaration of AI and AI-assisted technologies in the writing process

During the preparation of this work, the author used ChatGPT (OpenAI 5.2, San Francisco, CA, USA) to assist with troubleshooting of R code and with drafting, language editing, and revision of selected parts of the manuscript.

ChatGPT was not used to generate, analyze, or interpret any data or scientific content. All AI-assisted content was reviewed and edited by the author, who takes full responsibility for the content of the published article.

## Research funding

This research did not receive any specific grant from funding agencies in the public, commercial, or not-for-profit sectors.

## Declaration of competing interest

The authors declare that they have no known competing financial interests or personal relationships that could have appeared to influence the work reported in this paper.
