# Supplementary material for: Molecular sampling limits of ctDNA detection in clinical plasma samples
Source: J Liq Biopsy. 2026 Jul 4;13:100480. doi: 10.1016/j.jlb.2026.100480 (PMC13351401; doi:10.1016/j.jlb.2026.100480)
Supplement: Multimedia component 3 [file mmc3.docx]

**Supplementary Material**

**“Molecular Sampling Limits of ctDNA Detection in Clinical Plasma Samples”**

*The Journal of Liquid Biopsy*

**Supplementary Methods**

This supplement provides dataset-specific conversion procedures, the full Poisson-based sampling framework, sensitivity analyses for plasma volume and effective assay input, and implementation details for all analyses and visualizations.

**Genome equivalent distributions**

Genome-equivalent (GE) distributions were assembled from three independent liquid-biopsy datasets to capture real-world variability in analyzable cfDNA input. The analysis included 4,141 samples from a large pan-cancer clinical cohort reported by Jee et al. [12], 681 longitudinal plasma samples from 119 patients with early breast cancer reported by Elliott et al. [13], and 416 plasma samples from 355 individuals across multiple cancer and control cohorts reported by Widman et al. [14].

The datasets differed with respect to plasma volume and cfDNA reporting format, including cfDNA concentration normalized to plasma volume, values already expressed in genome equivalents, and total cfDNA mass recovered from defined plasma volumes. For comparability, all quantities were converted to haploid genome equivalents using 3.3 pg DNA per haploid genome (see Supplementary Table S1).

Datasets without directly comparable volume information (i.e. Jee et al. [12] and Widman et al. [14]) were standardized to 4 mL plasma equivalents as a pragmatic reference input consistent with commonly recommended minimum plasma volumes for routine cfDNA analysis [15]. Jee et al. [12] and Widman et al. [14] were standardized to a 4-mL plasma-equivalent (for Widman et al. using per-sample extracted DNA from NGS-based cfDNA analysis at 1–2 ml input per Supplementary Table S1). Elliott et al. [13] provided directly measured plasma volumes. Extracted GE from samples from Elliott et al. [13] had a median of 11,388 GE (interquartile range (IQR), 7,332–19,240 GE), derived from a mean plasma volume of 3.99 mL (median 4.03 mL; range 2.06–5.73 mL). After standardization to 4 mL plasma equivalents, the median GE was 4,220 GE (IQR, 2,429–9,498 GE) in Jee et al. [12] and 16,590 GE (IQR, 9,697–34,627 GE) in Widman et al. [14]. Overall, the combined dataset comprised 5,238 samples with a median of 5,531 GE and a range of IQR 2,784–13,060 GE (see Figure 1a: see also Supplementary Figure S1). GEs can be estimated from cfDNA concentrations using fluorometric, qPCR-based, or NGS-derived quantification methods, all of which are available in routine laboratory workflows [8, 15]. Each approach introduces its own measurement uncertainty, arising from non-specific DNA detection, amplification efficiency, or library preparation variability, respectively, which is not explicitly propagated in the present model. Users should therefore be aware that the true effective GE input may deviate from the measured value, compounding the sampling constraints described in the main text.

**Modeling expected mutant molecules**

Effective assay input was defined as N_eff_ = *N* × *f*, where N is the number of genome equivalents and *f* the fraction of extracted cfDNA entering the assay; *f* = 1 was assumed in the primary analysis. The expected number of mutant molecules at a given variant allele fraction (VAF) was modeled as *λ* = *N_eff_* × VAF. Under the assumption of random molecular sampling, mutant molecule counts were modeled using a Poisson distribution with mean *λ*. The probability of observing at least *k* mutant molecules was calculated as

$$P(X\geq k)=1-\sum_{i=0}^{k-1} \frac{e^{-\lambda}\lambda^{i}}{i!},$$

for *k* = 1, 3, 5, and 10. A Poisson-based lower limit of detection (LOD) was defined for each sample as the minimum VAF associated with a 95% probability of observing at least *k* mutant molecules. This LOD value was expressed as percent VAF. The modeling framework was intended to approximate input-limited detectability for single-locus, variant-specific alterations, particularly SNVs and indels. Other alteration classes, such as gene fusions or copy-number alterations, were not modeled explicitly because their detectability may depend on additional assay- and signal-specific factors beyond the simplified Poisson sampling assumptions used here. The implications of this Poisson-based model for required molecular input and plasma volume are illustrated in Supplementary Figure S3. To examine the dependence of the findings on the chosen reference plasma volume, supplementary sensitivity analyses were performed for theoretical plasma inputs of 1, 8, and 12 mL in addition to the primary 4 mL reference scenario. These analyses assumed proportional scaling of genome-equivalent input with plasma volume and were intended to illustrate how input-limited detectability shifts across lower- and higher-volume sampling scenarios (see Fig. 2). As a sensitivity analysis, reduced effective assay input fractions (f = 0.9, 0.6, and 0.1) were modeled in addition to the primary *f* = 1 scenario for the pooled 4 mL reference dataset. These scenarios were intended to represent progressively lower fractions of extracted cfDNA entering the analytical workflow, for example due to library preparation losses (viz. conversion yield cf. Song et al. [8]), but also assay input constraints, repeat testing, or allocation of material to parallel analyses. For each scenario, *N_eff_* = *N* × *f* was used to recalculate Poisson-based lower limits of detection and the proportions of samples achieving predefined sensitivity thresholds for k=1, 3, 5, and 10 mutant molecules (see Fig. 2).

**Web-based implementation of the sampling model**

To facilitate practical application of the model, an interactive Shiny web application (<https://aga1.shinyapps.io/ctdna_lod_app/>) based on the same Poisson framework described above was implemented. The app allows users to enter haploid genome equivalents directly or estimate them from cfDNA concentration and assay input volume, apply an effective input fraction (*f*), define the target detection probability, and evaluate standard or custom mutant-molecule thresholds (*k*). The tool returns sample-specific theoretical LOD estimates, detection probability curves across VAF ranges, and downloadable result tables and plots. The application is intended for research and educational use and is not designed for clinical decision-making. The web application source code has been included in the Supplementary Material.

**Supplementary Tables**

**Supplementary Table S1. Harmonized input data used for Poisson-based ctDNA detectability modeling.** The table summarizes the sample-level input data compiled from the three included liquid-biopsy studies and the corresponding harmonized genome-equivalent (GE) values used for the pooled analysis. Depending on the source study, original inputs were reported as cfDNA concentration normalized to plasma volume, total cfDNA mass, or genome equivalents. All values were converted to haploid genome equivalents using 3.3 pg DNA per haploid genome. Where required for comparability, GE values were standardized to 4 mL plasma equivalents as described in the Methods.

**Supplementary Figures**

**
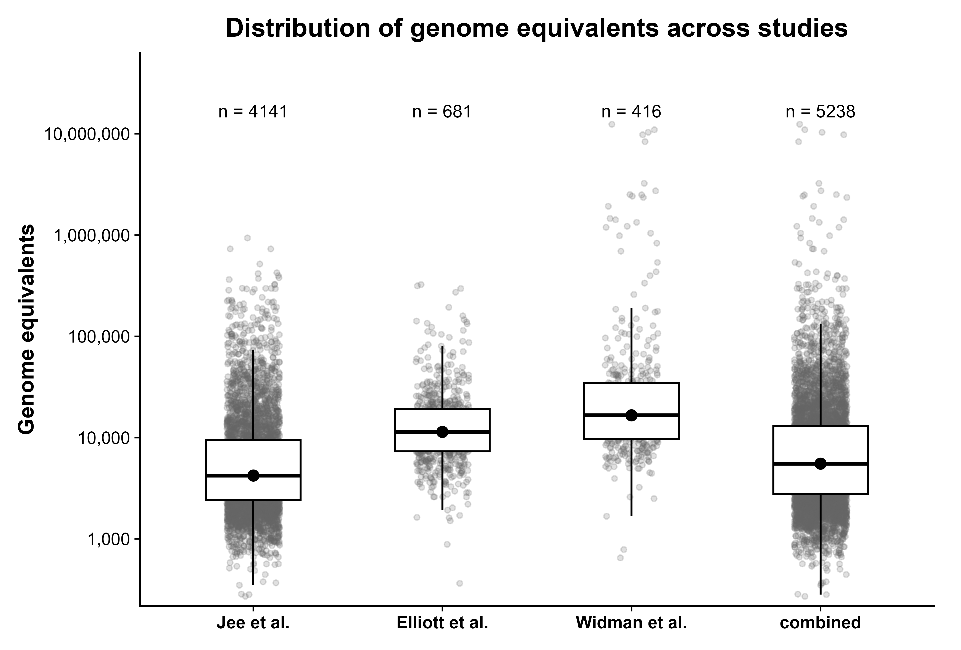
**

**Supplementary Figure S1. Genome equivalent distributions across studies.** Boxplot of genome equivalents (GE) per sample are shown for each study and for the combined dataset. Jittered points represent individual samples. The y-axis is plotted on a log10 scale. Sample numbers are indicated above each boxplot.

**
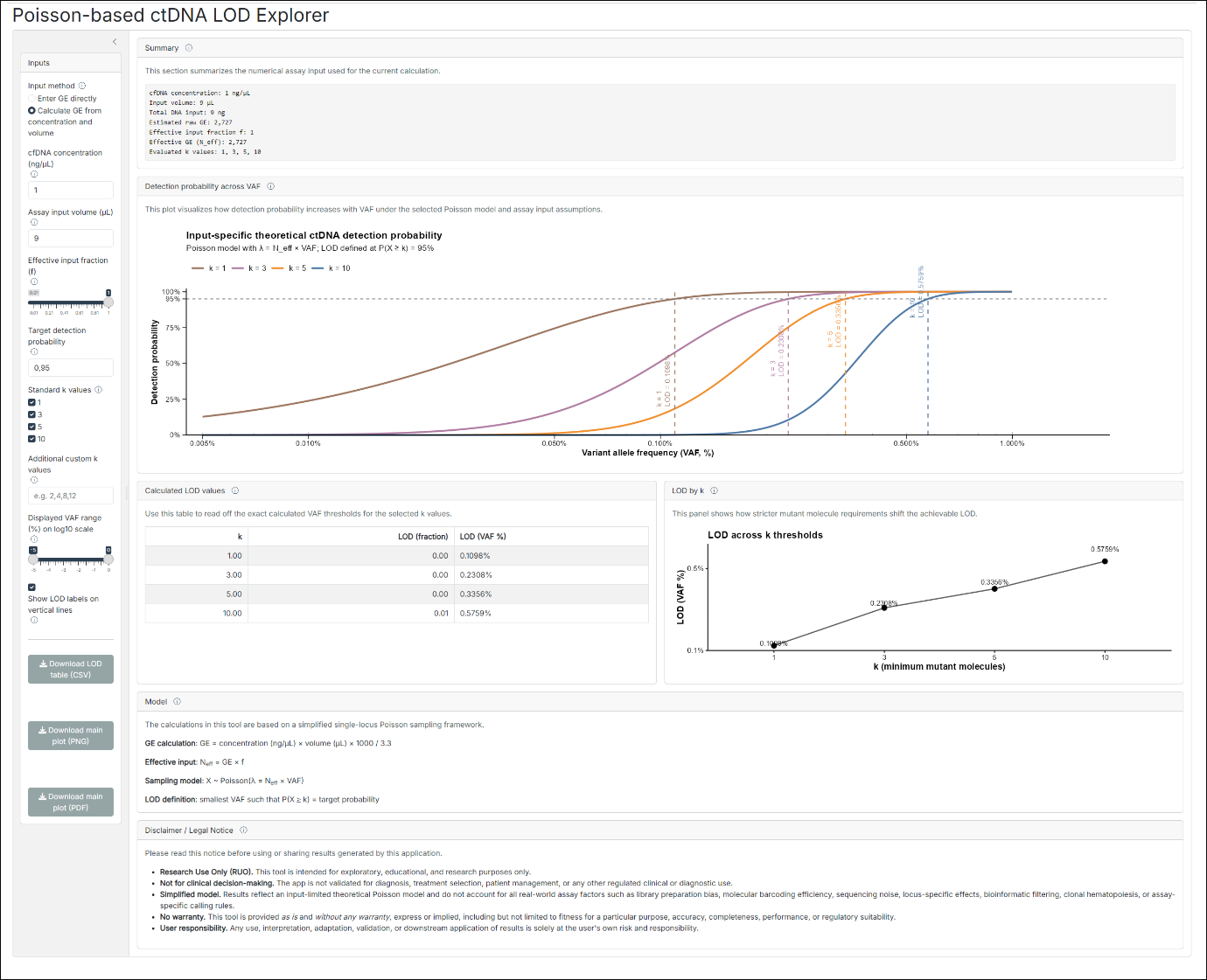
**

**Supplementary Figure S2. Example output of the Poisson-based ctDNA LOD Explorer** (<https://aga1.shinyapps.io/ctdna_lod_app/>). A web application designed to illustrate how cfDNA input constrains the theoretically achievable sensitivity of ctDNA assays under a Poisson sampling model. The app estimates the input-limited theoretical ctDNA limit of detection (LOD) from either directly entered haploid genome equivalents (GE) or from cfDNA concentration and assay input volume. In the example shown, 1 ng/µL cfDNA and 9 µL input correspond to an estimated 2,727 GE. The main panel displays the Poisson-based probability of detecting at least *k* mutant molecules across variant allele frequencies (VAFs) for *k* = 1, 3, 5, and 10, with the LOD defined as the VAF at which detection probability reaches 95%. Dashed vertical lines mark the corresponding LOD for each *k*, and the calculated values are additionally summarized in a table and a secondary plot.

**
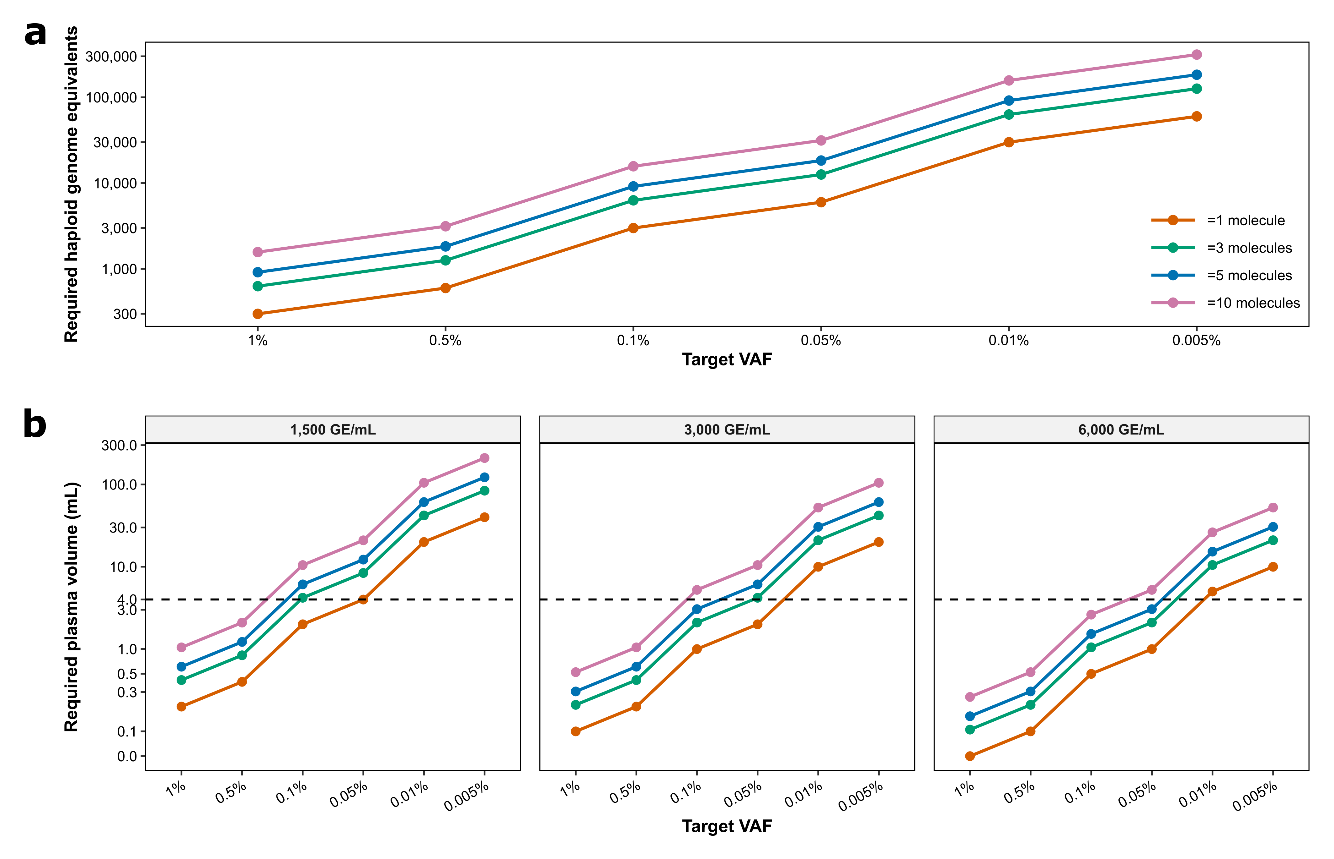
**

**Supplementary Figure S3. Plasma volume requirements for single-locus ctDNA detection under a Poisson sampling model. (a)** The number of haploid genome equivalents required to achieve a 95% probability of observing at least 1, 3, 5, or 10 mutant molecules was calculated for target VAFs ranging from 1% to 0.005%. **(b)** These genome-equivalent requirements were converted to required plasma volumes assuming cfDNA yields of 1,500, 3,000, or 6,000 haploid genome equivalents per mL plasma. The dashed line denotes a representative 4 mL plasma input. The model isolates stochastic sampling effects and does not account for additional losses arising from pre-analytical handling, extraction inefficiency, library preparation, or assay-specific analytical sensitivity.
